# Increased 5-HT2C receptor editing predisposes to PTSD-like behaviors and alters BDNF and cytokines signaling

**DOI:** 10.1038/s41398-019-0431-8

**Published:** 2019-02-21

**Authors:** Mathilde Règue, Corinne Poilbout, Vincent Martin, Bernard Franc, Laurence Lanfumey, Raymond Mongeau

**Affiliations:** 1 Inserm UMR S894, Centre de Psychiatrie et Neuroscience, Université Paris Descartes, 75014 Paris, France; 2 EA 4475, Pharmacologie de la circulation cérébrale, Université Paris Descartes, 75006 Paris, France

## Abstract

Post-traumatic stress disorder (PTSD) is a trauma- and stress-related disorder with dysregulated fear responses and neurobiological impairments, notably at neurotrophic and inflammation levels. Understanding the mechanisms underlying this disease is crucial to develop PTSD models that meet behavioral and neurobiological validity criteria as well as innovative therapeutic approaches. Serotonin 2C receptors (5-HT2CR) are known for their important role in anxiety, and mice having only the fully edited VGV isoform of 5-HT2CR, which thereby overexpressed brain 5-HT2CR, are of special interest to study PTSD predisposition. Innate and conditioned fear-related behaviors were assessed in VGV and wild-type mice. mRNA expression of brain-derived neurotrophic factor (BDNF), tissue-plasminogen activator (tPA), and pro-inflammatory cytokines (*IL-6*, *IL-1β*, and calcineurin*)* were measured by qRT-PCR. The effect of acute and chronic paroxetine was evaluated on both behavior and gene expression. VGV mice displayed greater fear expression, extensive fear extinction deficits, and fear generalization. Paroxetine restored fear extinction in VGV mice when administered acutely and decreased innate fear and fear generalization when administered chronically. In parallel, *Bdnf*, *tPA*, and pro-inflammatory cytokines mRNA levels were dysregulated in VGV mice. *Bdnf* and *tPA* mRNA expression was decreased in the hippocampus but increased in the amygdala, and chronic paroxetine normalized *Bdnf* mRNA levels both in the amygdala and the hippocampus. Amygdalar calcineurin mRNA level in VGV mice was also normalized by chronic paroxetine. VGV-transgenic mice displayed behavioral and neurobiological features that could be accessory to the investigation of PTSD and its treatment. Furthermore, these data point out to the role of 5-HT2CR in neuroplasticity and neuroinflammation.

## Introduction

Post-traumatic stress disorder (PTSD) is a prevalent trauma- and stress-related disorder caused by exposure to a strong psychological trauma. This disorder is characterized by hyperarousal and dysregulated fear responses triggered by contexts and cues reminding the traumatic event. PTSD patients also suffer from fear memory extinction deficits and contextual fear generalization^1,2^. Chronic treatment with selective serotonin reuptake inhibitors (SSRIs, such as paroxetine) is the first-line pharmacological approach, while behavioral therapies include the prolonged exposure therapy that generates fear extinction. However, no more than 30% of patients reached full remission with pharmacological therapies, while at least 40% of patients are non-responders to behavioral approaches^1,2^. Combining pharmacological and prolonged exposure therapies could theoretically present increased benefits. Nevertheless, clinical studies on cognitive behavioral therapy and SSRI are sparse and non-conclusive^3,4^. There are indications that chronic antidepressant treatment may in some cases even impair fear extinction^5^.

A number of reports argue for the involvement of 5-HT and in particular serotonin 2c receptors (5-HT2CR) in anxiety. PTSD patients display a range of serotonergic abnormalities, including an exaggerated stress response to the anxiogenic 5-HT2CR agonist meta-chlorophenylpiperazine^6^ and typical traits of a serotonergic alteration including irritability, aggression, impulsivity, and suicidability^7^, which are themselves associated with upregulation of 5-HT2CR and altered 5-HT2CR mRNA splicing/editing^8–10^.

Animal models such as predator or aggressive conspecific exposure, or the single prolonged stress exposure, provided some understanding about the pathophysiology of PTSD^11,12^. These models create anxiety-like behaviors as well as alterations of brain-derived neurotrophic factor (BDNF)-TrkB and serotonergic receptors. Stresses triggering PTSD-like states increase the expression of brain 5-HT2CR. PTSD symptoms may be alleviated by antidepressant drugs with 5-HT2CR antagonist properties^13–15^ or by selective 5-HT2CR antagonists^16–18^. Notably agomelatine, an antidepressant with melatonergic agonist and 5-HT2C antagonist properties, is now considered as a possible compound for the treatment of anxiety disorders including PTSD as it alleviates anxiety symptoms in animal models^19,20^ and in humans^21^ while presenting a good tolerability profile in patients^21^.

The 5-HT2CR is among the most frequently pinpointed for its implications in anxiety, stress, and fear behaviors^22–25^. It is the only serotonergic receptor undergoing adenosine-to-inosine edition of its pre-mRNA. Maternal separation stress, generating PTSD-like predispositions, robustly increased 5-HT2CR editing^26^. We have shown that increasing 5-HT2CR editing level interferes with 5-HT2CR mRNA alternative splicing processes, leading to a large upregulation of the receptor at cell membrane^24^. Dysregulation of 5-HT2CR editing using mice expressing only the fully edited VGV isoform of the 5-HT2CR (VGV mice) enhanced anxiety, aggression, and innate fear behaviors^24,27^. We thus determined here if VGV mice could display additional features of PTSD in a conditioned fear paradigm.

Chronic PTSD is also associated with changes in biological markers, including BDNF and pro-inflammatory cytokines, the blood levels correlating with SSRI effectiveness^28,29^. 5-HT2CR blockade or deletion both alter the expression of BDNF^30,31^. This neurotrophin is a key regulator of synaptic plasticity and behaviors, while BDNF–serotonergic interactions appear to occur in anxio-depressive disorders^32^. BDNF is also known to regulate cortical, hippocampal, and amygdalar-dependent memories^33^, while the BDNF/TrkB pathway has been linked to fear conditioning processes^34^, fear extinction^35,36^, and fear generalization^37^. We thus focused on *Bdnf* and also the mRNA encoding *tPA*, which mediates the conversion of precursor proBDNF into mature BDNF. Furthermore, considering the important crosstalks between serotonin, BDNF, and inflammation^38^, as well as cytokine-5-HT2CR editing interactions^39^, we also examined brain levels of IL-6, IL-1β, and calcineurin in VGV mice. It has been proposed that a mutual feedback loop regulation involving the serotonin transporter and BDNF helps in maintaining the brain balance between serotonergic and neurotrophin signaling^38^. The cytokine-induced perturbations of brain serotonergic activity may also alter the BDNF/TrkB pathway^38^.

The objectives of this work were to define the consequence of the 5-HT2CR editing modification on fear behaviors, to pinpoint the involvement of serotonin and 5-HT2CR on these outcomes and on the downstream BDNF and inflammation pathways and finally to examine the effects of paroxetine treatments on behavioral and neurobiological changes found in VGV mice. We focused on paroxetine, the first-line antidepressant drug treatment for PTSD, which does not have affinity for 5-HT2CR, and which is known to desensitize 5-HT2CR after chronic treatment^40^.

## Methods and materials

### Animals

Ten-week-old male, either control C57BL/6J or expressing VGV 5-HT2CR, mice were used, unless detailed. Details are given in the Supplementary Materials. All procedures concerning animal care and treatment were carried out in accordance with protocols approved by French ethical committee #C2EA-05 Charles Darwin and licensed by Directorate General for Research and Innovation (French government), under protocol authorization #00966.02. The experimental groups were randomly designed.

### Experimental design

For behavioral studies using chronic paroxetine, experimental designs are described in Fig. 2. For behavioral studies using acute paroxetine, experimental designs are described in Fig. 3. For Reverse transcription quantitative polymerase chain reaction (RT-qPCR) analyses, cerebral structures of interest were collected from dedicated groups after treatment.

### Conditioned and innate fear procedures

To observe the consequences of the VGV genotype on innate and conditioned fear behaviors, we performed ultrasound-induced fear evaluation and fear conditioning experiments.

Apparatus and analysis are detailed in Supplementary Materials. Behaviors were monitored by a video camera, and freezing, defined as total lack of movement except respiration, was scored.

Fear conditioning: On Day 1, mice were placed in the conditioning chamber and after a 3-min baseline period, they received six times an auditory conditional stimulus (CS; 30 s, 2.5 kHz, 85 dB) immediately followed by the unconditioned stimulus (US; 2 s, 0.5 mA foot-shock, inter-trial intervals 2 min).

Cued extinction: On Day 2, for experimental designs 1 and 3, or Day 30, for experimental design 2 (Fig. 2a, f), mice received 20 exposures to the same tone (30 s, 2.5 kHz, 85 dB; inter-trial intervals 5 s) in a new context to assess CS-induced fear and its extinction. As shown in Fig. 3, the same procedure was repeated on Day 3, to determine the consolidation of the fear extinction memory.

The mice were tested for innate fear reactions to trains of ultrasonic stimuli (100 ms frequency sweeps of 17–20 kHz, 85 dB, alternately 2 s off and 2 s on) in their home cage during 1 min and after a 3-min baseline period, as previously described^24^. Data were monitored by a video camera. Innate fear corresponds to the immediate expression of reflex-like defensive behaviors, here freezing, generated by a brief stimulus not associated with a previous aversive event.

### Barnes maze test

Tests were performed as previously described^41^. Learning and reversal learning were each performed during 4 days with three sessions per day. Errors and latencies before finding the escape box were measured. The reversal probe test was conducted immediately after the last reversal learning session.

### Quantification of RNA levels by RT-qPCR

To determine the role of serotonin and the 5-HT2CR on the BDNF/TrkB and inflammation pathways, we quantified the mRNA expression of key molecules of these pathways.

Tissue samples were quickly removed and frozen in liquid nitrogen. Total mRNA was extracted using TRI Reagent (Ambion, Applied Biosystems, Courtaboeuf, France), following manufacturer’s instructions. Reverse-transcription was performed using High Capacity cDNA Reverse Transcription kit (Applied Biosystems, Courtaboeuf, France) and PCR amplifications were performed using a SYBR Green mix (KAPA SYBR Fast qPCR Master Mix, KAPA Biosystems, MA, USA). For detailed cycling protocols and primers’ sequences, see supplementary information.

### Drug administrations

To assess the effect of SSRI on our different targets, we performed chronic and acute administrations of paroxetine, as well as acute administration of 5-HT2C antagonists.

For chronic treatment, paroxetine HCl (Sequoia Research Products, Pangbourne, UK) was administered in drinking water (~5.5 mg/kg/day, starting 4 weeks before the experiments), to avoid stress sensitizing VGV mice with the daily injection stress. Chronic treatment was not interrupted during behavioral studies. Treatment intake was closely monitored and the solution concentration was adjusted per animal, to ensure equivalent intake of paroxetine. Acute paroxetine HCl was injected intraperitoneally (i.p.) at 16 mg/kg (in 0.9% saline). For both paroxetine behavioral studies, experimental timeline is described in Figs. 2 and 3. SB242084 (Abcam Biochemicals, Cambridge, UK) was injected i.p. at 1 mg/kg (in 0.9% saline, 1% Tween 80). Agomelatine (Servier Laboratories, Suresnes, France) was injected i.p. at 50 mg/kg and dissolved in 1% hydroxyethylcellulose (Servier Laboratories).

### Statistical analysis

The number of animals per experiment was based on a power analysis^42^. Data were analyzed using Prism (GraphPad, San Diego, USA). For two groups comparisons, an unpaired Student’s *t*-test was used, with Welch’s correction if needed. All remaining data were compared using a two-way analysis of variance (ANOVA), followed by the Bonferroni post-hoc test. See Supplementary section for details. Data are presented as mean ± s.e.m.

## Results

### The conditioned fear-related behaviors of VGV mice

**Fig. 1:**
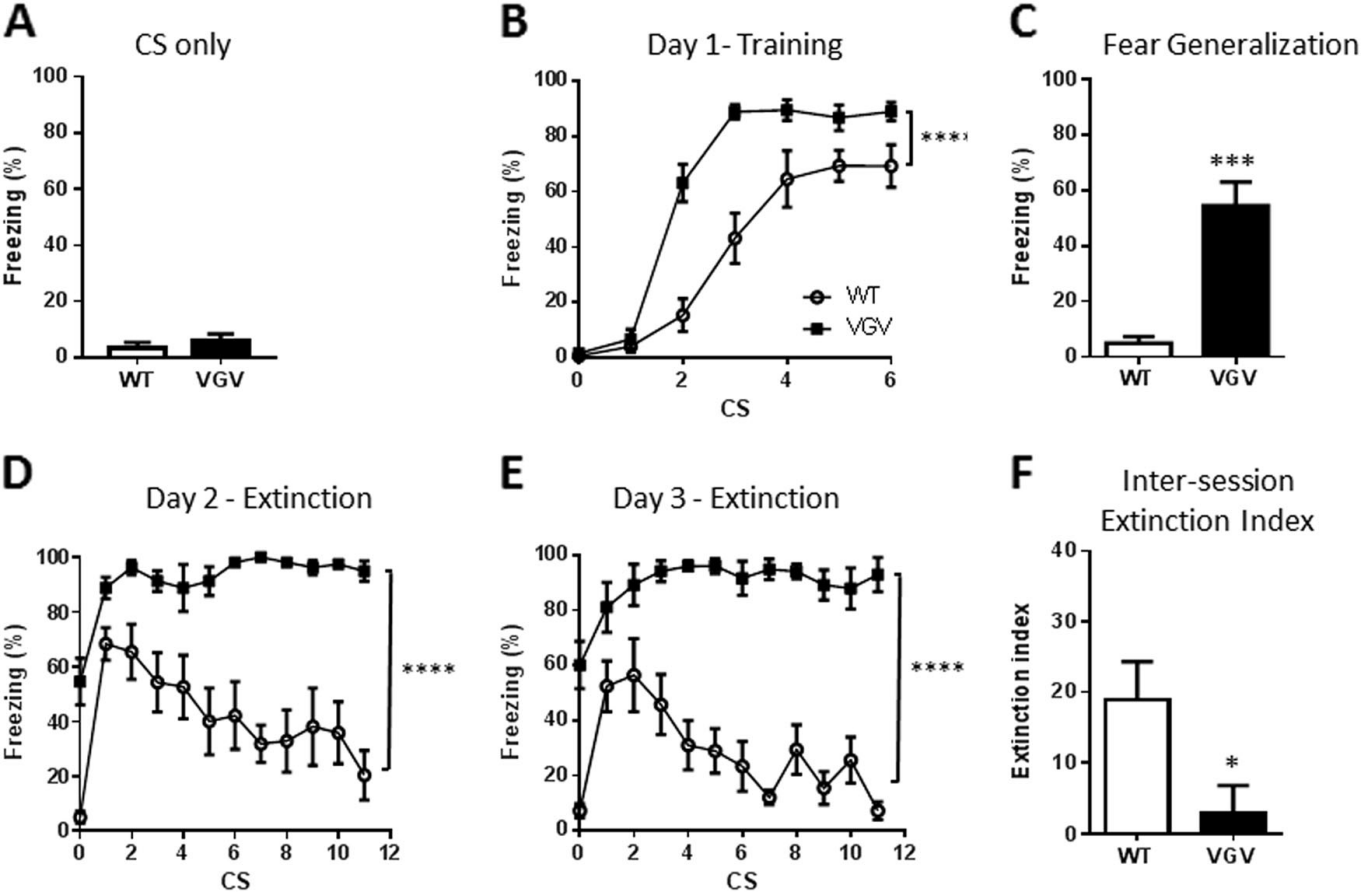
Alteration of conditioned fear behaviors in VGV mice. **a** The sound used as CS (30 s, 85 dB, 2.5 kHz) for the conditioning is neutral and did not elicited fear responses in VGV mice when presented alone. **b** VGV mice acquired fear conditioning faster compared to WT mice. Mean ± s.e.m percent of time spent freezing during each CS presentation. Two-way ANOVA with repeated measures indicated a significant effect of Genotype [*F* (1, 16) = 28.42, *p* < 0.0001], Time [*F* (6, 96) = 107.9, *p* < 0.0001], and an interaction between those factors [*F* (6, 96) = 7.85, *p* < 0.0001]. **c** VGV mice displayed high fear generalization during the baseline period of the tone-testing session (*t*_10.31_ = 5.63, *p* = 0.0002). **d** Throughout the CS presentation, VGV mice presented very high freezing with a lack of fear extinction and a generalization of fear at *t* = 0. For the first extinction session, a two-way ANOVA with repeated measures indicated a significant effect of Genotype [*F* (1, 16) = 70.82, *p* < 0.0001], Time [*F* (12, 192) = 9.56, *p* < 0.0001], and an interaction between factors [*F* (12, 192) = 3.51, *p* = 0.0001]. **e** Twenty-four hours later, a second extinction session was conducted. Two-way ANOVA with repeated measures indicated a significant effect of Genotype [*F* (1, 16) = 117.8, *p* < 0.0001], Time [*F* (12, 192) = 6.21, *p* < 0.0001], and an interaction between factors [*F* (12, 192) = 4.51]. **f** WT mice displayed retention of the extinction memory, quantified by an extinction index defined as (Day 2 freezing at CS1) − (Day 3 freezing at CS1), while VGV did not (*t*_15_ = 2.45, *p* = 0.0271); *p < 0.05, ***p < 0.001, ****p < 0.0001 vs. WT

We first assessed the emotional neutrality of the auditory cue in transgenic mice. Mice were placed in the fear conditioning apparatus and submitted to the CS only. The 2.5 kHz tone did not a priori elicit any fear responses in VGV mice (Fig. 1a). Furthermore, on Day 1 of the fear conditioning procedure, similar to wild type (WT), VGV mice did not show freezing during baseline or in reaction to the first tone (Fig. 1b). Freezing progressively increased during the first five CS deliveries to reach ~70% in WT mice, but VGV mice reached this plateau at the third CS delivery.

Extinction was induced by repeated CS exposure in a new context. At both Days 2 and 3, VGV mice exhibited a high baseline freezing, indicative of contextual fear generalization (freezing at Day 2: 54.6 ± 8.5%, Day 3: 60.2 ± 8.6%). On Day 2, the difference in baseline freezing in VGV mice compared to WT mice was as much as 50% (Freezing WT: 2.1 ± 1%, VGV: 54.6 ± 8.5%; Fig. 1c). Freezing decreased in WT mice with repeated CS, without shocks, to reach 20.4 ± 9.1%, while it remained maximal in VGV mice (Fig. 1d, e). Retention of extinction between the two days was quantified by analyzing an inter-session extinction index defined as (Day 2 freezing at CS1) − (Day 3 freezing at CS1), for each mouse, and indicated a significant reduction of extinction in VGV mice (Fig. 1f). Contrary to WT mice, there was no reduction in the total amount of freezing at Day 3 vs. Day 2 in VGV mice (WT = −15.3 ± 5.2% vs. VGV = −2.2 ± 2.9%; *t*_15_ = 2.12, *p* = 0.033). Considering that *Htr2c* is an X-linked gene^43^, we assessed whether gender differences existed, but there were none (Fig. S1).

Even 1 month after conditioning, deficits in fear extinction and fear generalization persisted (Fig. S2A, B). VGV mice also showed a deficit of context extinction (Fig. S2C) persisting even after 6 days of re-exposure (data not shown).

### Spatial memory and cognitive flexibility in VGV mice

It was necessary to assess whether VGV mice have extended memory alterations beside those observed in fear conditioning. No difference was found in the Barnes maze (see supplementary data, Fig. S3). Furthermore, VGV mice could perform this test optimally as they did not present any locomotor impairment (Fig. S3C, S3F).

### Assessment of paroxetine brain delivery

To validate the efficacy of oral chronic paroxetine treatment, corticotropin-releasing hormone (Crh) mRNA level was measured, as antidepressant treatments were found to reduce the *Crh* gene expression in rodents^44^. *Crh* mRNA was decreased to the same amplitude in the brain of WT and VGV mice (Fig. S4).

### Effects of chronic paroxetine on conditioned and innate fear

**Fig. 2:**
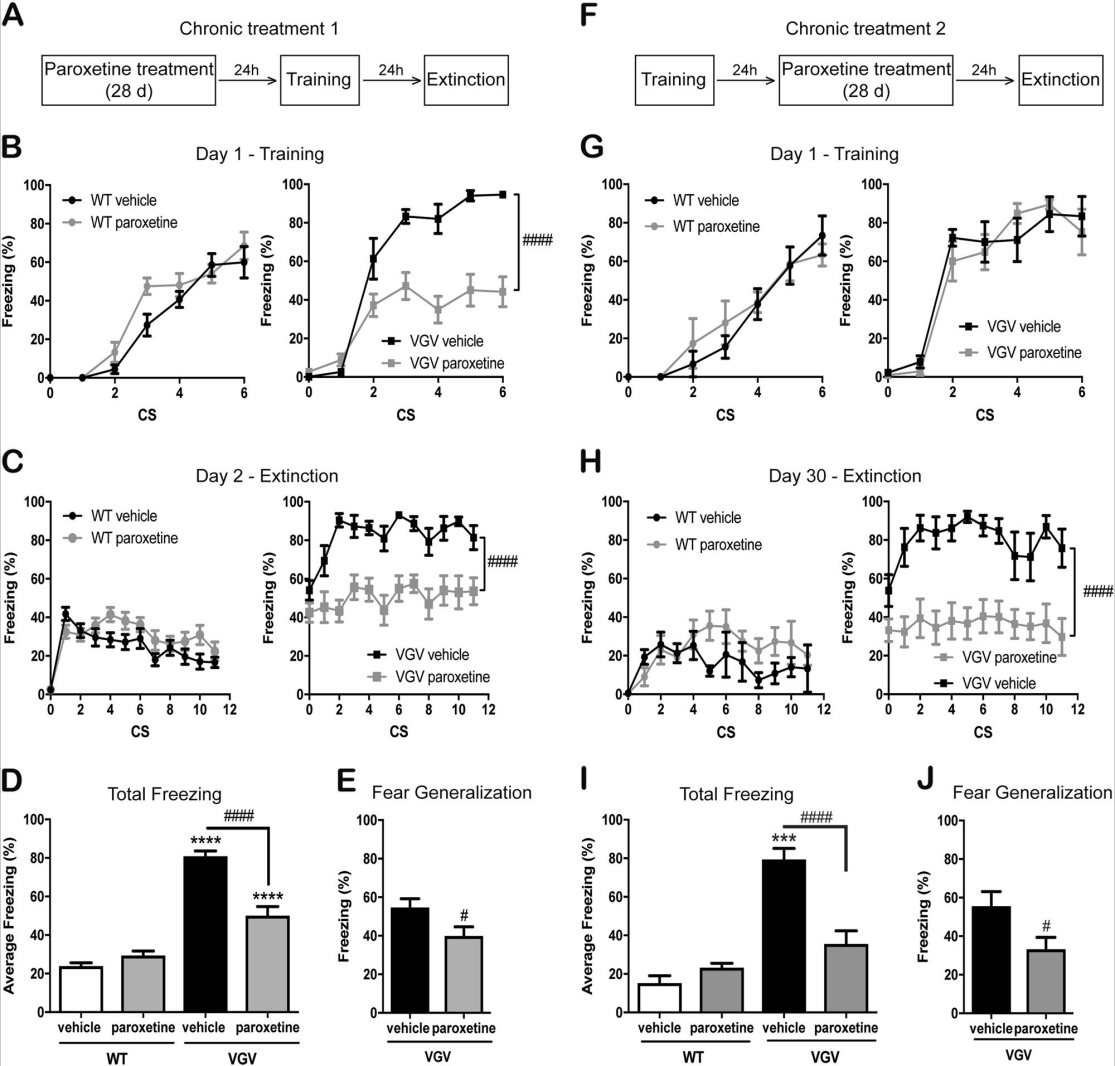
Effects of chronic paroxetine on fear behaviors in VGV mice. For chronic paroxetine studies, groups underwent per os treatment (~5.5 mg/kg/day) for 28 days in drinking water and two different timelines of experiment were used. In each design, chronic treatment was not interrupted during the whole set of behavioral studies. For each experiment, only the 11 first CS are presented as, after a prolonged time, quiet immobility may be confounded with freezing. **a** In the first design, chronic treatment was given for 28 days prior to the fear conditioning, which started on the 29th day. **b** When administered before the acquisition session, chronic paroxetine decreased freezing in VGV mice during the CS presentation of the acquisition phase. In WT mice, two-way ANOVA indicated a significant effect of Time [*F* (6, 96) = 77.94, *p* < 0.0001] but no effect of treatment. In VGV mice, two-way ANOVA indicated a significant effect of Treatment [*F* (1, 20) = 33.85, *p* < 0.0001], Time [*F* (6, 120) = 63.73, *p* < 0.0001], and an interaction between factors [*F* (6, 120) = 11.32, *p* < 0.0001]. **c** Chronic paroxetine tended to impair extinction in WT mice. ANOVA indicated a nearly significant effect of Treatment [*F* (1, 31) = 3.64, *p* = 0.06], Time [*F* (11, 341) = 14.81, *p* < 0.0001], and an interaction between factors [*F* (11, 341) = 1.89, *p* = 0.03], while in contrast reducing overall freezing in VGV mice. ANOVA indicated a significant effect of Treatment [*F* (1, 19) = 25.98, *p* < 0.0001] and Time [*F* (11, 209) = 4.32, *p* < 0.0001] but no interaction. **d** When analyzing the mean total freezing during the extinction session, there was a significant effect of Genotype [*F* (1, 50) = 168, *p* < 0.0001], Treatment [*F* (1, 50) = 17.94, *p* < 0.0001], and an interaction between factors [*F* (1, 50) = 36.78, *p* < 0.0001]. **e** Fear generalization is reduced by chronic paroxetine in VGV mice (*t*_19_ = 2.21, *p* = 0.04). **f** In the second design, chronic treatment was started 24 h after the training day (Day 1) of the fear conditioning paradigm. The 28-day treatment was followed by the extinction session, which consequently took place on Day 30 of the timeline. **g** In the experimental design 2, no difference was detected in the acquisition of the fear conditioning among each genotype, prior to treatment. **h** When chronic paroxetine is administered between the acquisition and the extinction sessions, there was a significant effect of Genotype [*F* (1, 24) = 35.89, *p* < 0.0001], Treatment [*F* (1, 24) = 7.88, *p* = 0.0098], and an interaction between factors [*F* (1, 24) = 16.54, *p* = 0.0004] for the mean total freezing during the extinction session. **i** Fear generalization is reduced by chronic paroxetine in VGV mice (*t*_17_ = 2.28, *p* = 0.0357). **j** Chronic paroxetine reduced the overall freezing in VGV mice. In WT mice, ANOVA indicated no effect of Treatment [*F* (1, 8) = 3.46, *p* = 0.1], a significant effect of Time [*F* (11, 88) = 2.35, *p* = 0.0138], and no interaction between factors [*F* (11, 88) = 1.01, *p* = 0.44]. In VGV mice, ANOVA indicated a significant effect of Treatment [*F* (1, 16) = 23.08, *p* = 0.0002] and Time [*F* (11, 176) = 2.44, *p* = 0.0074] but no interaction. ***p < 0.001, ****p < 0.0001 vs. WT Vehicle; #*p* < 0.05, ####*p* < 0.0001 vs. VGV Vehicle

We first performed a chronic paroxetine treatment prior to fear conditioning, as this was shown to desensitize 5-HT2CR^45^ (see experimental design Fig. 2a). Paroxetine-treated VGV mice displayed reduced freezing during the acquisition phase of fear conditioning compared to vehicle-treated VGV mice (Fig. 2b). Note that freezing reactions during fear acquisition in paroxetine-treated VGV mice were not increased compared to WT mice (mean freezing calculated on the whole acquisition session for each group. Vehicle-treated WT: 27.3 ± 10%; vehicle-treated VGV: 59.7 ± 15.6%; paroxetine-treated VGV: 30.9 ± 6.7%; Fig. 2b). During extinction, a global decrease in freezing was observed in paroxetine vs. vehicle-treated VGV mice. However, the extinction deficit remained in paroxetine-treated VGV mice (Fig. 2c). Paroxetine reduced the total amount of freezing during repeated CS presentation from 80.8 ± 2.7% to 50 ± 4.8% and decreased from 54.6 ± 4.6% to 39.9 ± 4.7% the freezing during baseline (contextual generalization) in VGV mice (Fig. 2d, e; WT mice had no generalization).

Using experimental design 2 (Fig. 2f), we determined the effect of chronic paroxetine on extinction once the training phase was completed, to mimic post-trauma paroxetine treatment in patients. First, we verified that groups from the same genotypes were not different before treatment during the acquisition session (Fig. 2g). Again, when measured 24 h after the end of paroxetine treatment, a global decrease in freezing (from 79.4 ± 5.7% to 35.5 ± 6.9%) and in fear generalization (Fig. 2j) occurred in paroxetine vs. vehicle-treated VGV mice while their extinction deficit remained (Fig. 2h, i).

We had previously shown that VGV mice display higher freezing to an innately aversive ultrasound delivered in their home cage^24^. VGV mice exhibited high freezing (43.9%) during the 1-min post-stimulus period, while WT mice displayed very little freezing during this period (Fig. S5). Chronic paroxetine effectively reduced ultrasound-induced freezing in VGV mice (−19.4 ± 8.7%; Fig. S5).

All these data suggest that chronic paroxetine induced an anxiolytic-like effect without restoring fear extinction in VGV mice.

### Behavioral effects of acute paroxetine

**Fig. 3:**
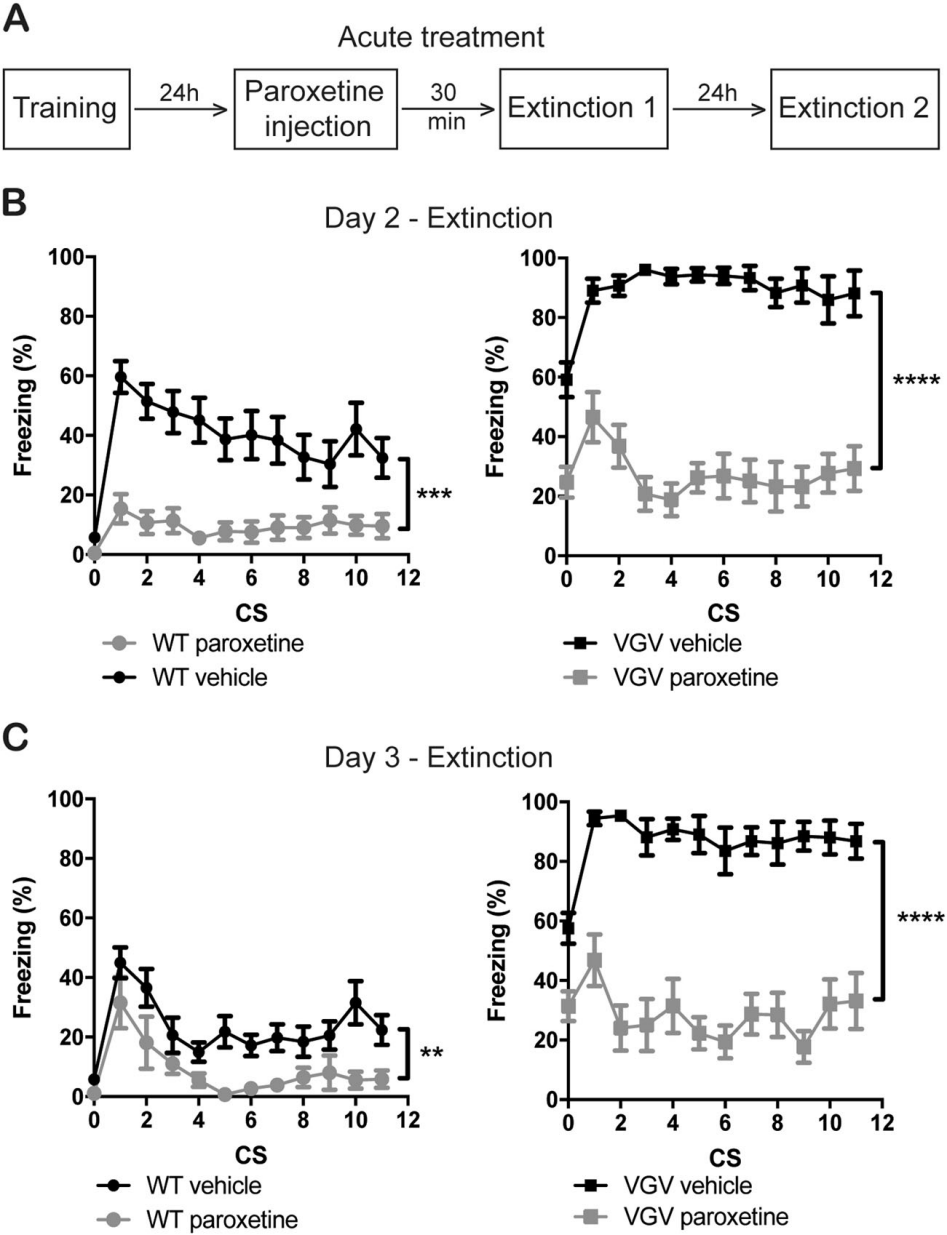
Effects of acute paroxetine on fear behaviors in VGV mice. **a** We assessed the effect of an acute injection of paroxetine on the extinction process. Paroxetine was injected intraperitoneally 24 h after the conditioned fear acquisition session and 30 min before the first extinction session. A drug-free extinction session was also performed 24 h later. **b** Acute paroxetine (16 mg/kg, i.p., 30 min before the first extinction session) strongly decreased freezing in WT mice (effect of Treatment [*F* (1, 20) = 21.44, *p* = 0.0002], effect of Time [*F* (11, 220) = 6.26, *p* < 0.0001], interaction between factors [*F* (11, 220) = 2.75, *p* = 0.002]) and decreased freezing during extinction in VGV mice (effect of Treatment [*F* (1, 23) = 135.0, *p* < 0.0001], effect of Time [*F* (11, 253) = 3.79, *p* < 0.0001], interaction between both factors [*F* (11, 253) = 3.96, *p* < 0.0001]). In addition, in the paroxetine-injected VGV mice, CS-induced freezing was similar to that of vehicle-treated WT mice (no effect of treatment, *F* (1, 22) = 2.59, *p* = 0.12). **c** After a 24-h period, VGV mice treated on the previous day with paroxetine presented extinction (effect of Treatment [*F* (1, 22) = 99.52, *p* < 0.0001], effect of Time [*F* (11, 242) = 3.02, *p* = 0.0008], interaction between factors [*F* (11, 242) = 2.84, *p* = 0.002]) while paroxetine-treated WT mice presented reduced freezing (effect of Treatment [*F* (1, 20) = 10.88, *p* = 0.0036], effect of Time [*F* (11, 220) = 9.89, *p* < 0.0001], but no interaction). Moreover, there was no difference in freezing between VGV mice treated on the previous day with paroxetine and vehicle-treated WT mice (no effect of treatment, *F* (1, 22) = 0.93, *p* = 0.34). Finally, the extinction index was significantly increased in paroxetine-treated VGV mice (*F* (3, 40) = 4.67, *p* = 0.0069, with Bonferroni post-hoc test indicating a significant difference between WT Vehicle and VGV Vehicle groups as well as between VGV Vehicle and VGV Paroxetine groups); ****p < 0.0001 vs. Vehicle

Because paroxetine desensitizes autoreceptors during chronic treatments, and somehow delayed here fear extinction in WT mice (Fig. 2c, h), we decided to assess the effect of an acute treatment (Fig. 3a) in conditions similar to antidepressant behavioral-screening assays (16 mg/kg, i.p., 30 min before test). Acute paroxetine decreased the expression of freezing during the extinction in both WT and VGV mice (Fig. 3b). In addition, it induced a significant progressive decrease of freezing within session in VGV mice (Fig. 3b, Fig. S6), as quantified by significant differences in the intra-session extinction index (Fig. S6A). After a 24-h period, a second extinction session was conducted (without paroxetine injection; Fig. 3a). Extinction was still observed within the session in VGV mice previously administered with paroxetine (Fig. 3c and Fig. S6B). However, fear extinction was not consolidated between Days 2 and 3 in VGV mice, as opposed to the WT vehicle group (inter-session extinction index, Fig. S6C). Nevertheless, acute paroxetine at D2 exerted also a decrement of fear generalization, and this decrement appeared consolidated at D3 (D2: *t*_22_ = 4.32, *p* = 0.0003; D3: *t*_22_ = 3.62, *p* = 0.0015; Fig. 3b, c at CS 0).

### Behavioral effects of 5-HT2CR antagonists

Administration of the selective and potent 5-HT2CR antagonist SB242084 (1 mg/kg, i.p.) also strongly inhibited freezing in VGV mice during the extinction process (Fig. S7A). However, SB242084 produced great hyperactivity in VGV mice, as might have been expected from the literature on the effect of SB242084 in WT mice^46^. To avoid this confounding effect, a less potent 5-HT2CR antagonist, the antidepressant compound agomelatine (50 mg/kg, i.p.), was used. Acute administration of agomelatine in VGV mice only tended to decrease both the total amount of freezing during the extinction session (Fig. S7B) and fear generalization (Fig. S7C) and did not favor any extinction in VGV mice (data not shown).

### Alterations of *Bdnf* mRNA expression in VGV mice

**Fig. 4:**
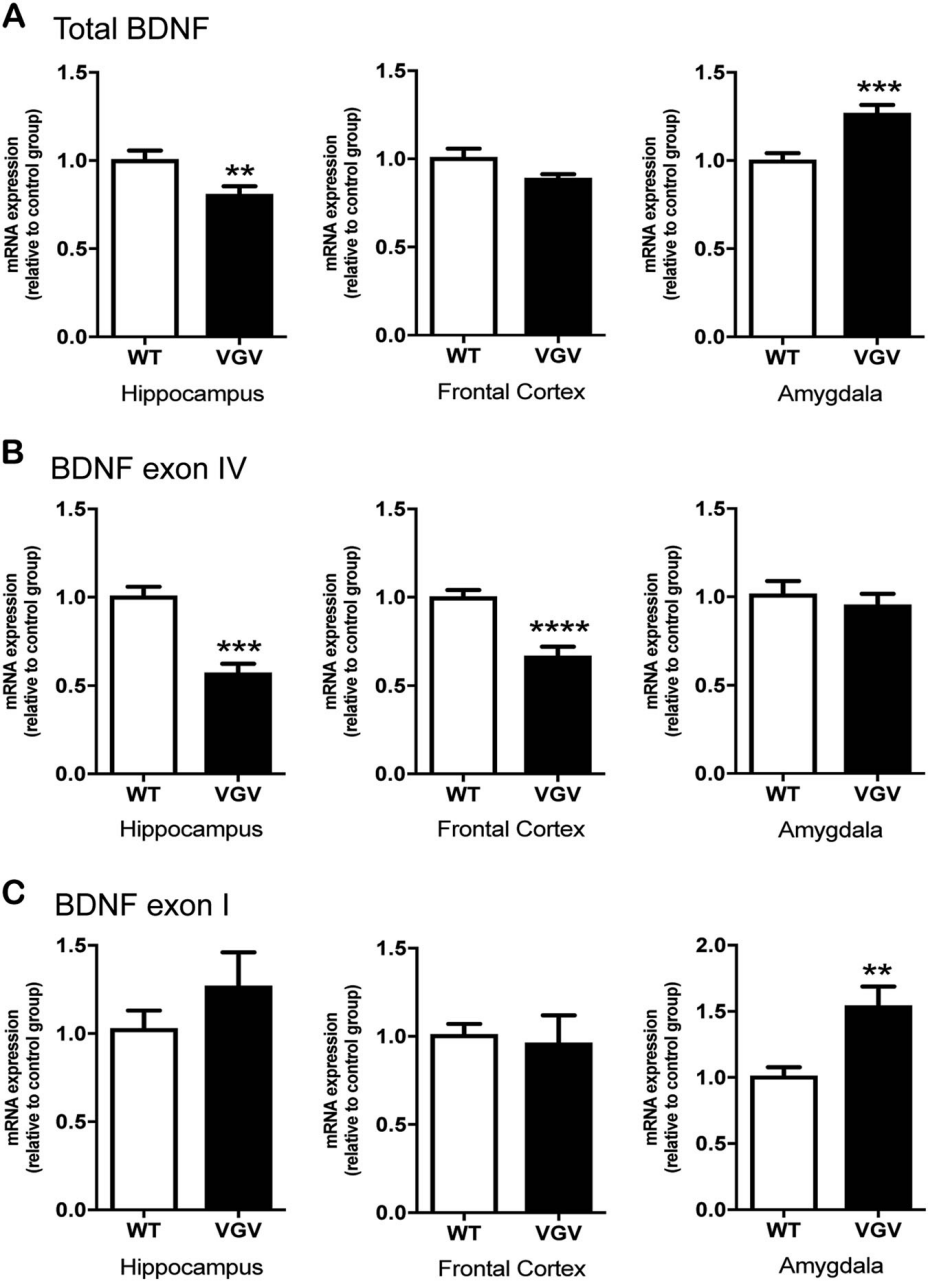
Basal *Bdnf* mRNA expression profile in the hippocampus, the frontal cortex, and the amygdala of VGV mice. **a**, **b** Total *Bdnf* and exon IV mRNA levels were significantly decreased in the hippocampus of VGV mice (Total *Bdnf*; *t*_14_ = 3.04, *p* = 0.009; Exon IV: *t*_14_ = 6.17, *p* < 0.0001). No difference was detected in exon I. **b** There were no significant changes in the frontal cortex in total *Bdnf* and exon I mRNA expression, although *Bdnf* exon IV mRNA expression was significantly decreased in VGV mice (*t*_14_ = 5.55, *p* < 0.0001). **a**, **c** Total *Bdnf* and exon I mRNA levels were significantly increased in the amygdala of VGV mice (Total *Bdnf*; *t*_15_ = 4.59, *p* = 0.0004; Exon I: *t*_9.69_ = 3.39, *p* = 0.007)

Basal expression of *Bdnf* mRNA was explored in several brain areas. The *Bdnf* gene is formed of nine exons, with the coding region located in exon IX corresponding to total *Bdnf*. *Bdnf* transcription occurs with various patterns of exons but we focused our analysis on exons I and IV because they are the main exons reported to be modulated in response to stress^47^, fear^48,49^, and neuronal activity^50^. *Bdnf* was decreased in the hippocampus (Fig. 4a, left), and there was also a tendency for a decrease in the frontal cortex (*t*_14_ = 1.86, *p* = 0.08; Fig. 4a, middle) of VGV mice. In contrast, *Bdnf* was increased in the amygdala of VGV mice (Fig. 4a, right). *Bdnf* exon IV was decreased in the hippocampus (Fig. 4b, left) and the frontal cortex (Fig. 4b, middle), and *Bdnf* exon I was increased in the amygdala of VGV mice (Fig. 4c, right).

### Effects of chronic paroxetine on trophic and inflammation factors in VGV mice

We examined the effect of chronic paroxetine in areas where total *Bdnf* was altered in VGV mice. In water-treated VGV mice, *Bdnf* mRNA expression was found decreased in the hippocampus (*t*_16_ = 3.92, *p* = 0.0012) and increased in the amygdala (*t*_13_ = 2.85, *p* = 0.0136; Table 1A). After chronic paroxetine, no difference in total *Bdnf* mRNA expression was detected between WT and VGV mice (Table 1A). In a separate analysis, we observed that chronic paroxetine normalized *Bdnf* mRNA in paroxetine-treated VGV mice compared to vehicle-treated mice, as it decreased amygdalar *Bdnf* mRNA and tended to increase hippocampal *Bdnf* mRNA (*t*_14_ = 2.55, *p* = 0.02 and *t*_12_ = 1.93, *p* = 0.08, respectively; data not shown).

**Table 1:** Effects of chronic paroxetine on trophic and inflammation factors in VGV mice

	Vehicule			Paroxetine		
	WT		VGV	WT		VGV
**A Hippocampus**						
BDNF pathway						
Total *Bdnf*	1.01 ± 0.05	0.77 ± 0.03	**	1.01 ± 0.04	0.87 ± 0.08	n.s.
*Bdnf* exon IV	1.02 ± 0.08	0.61 ± 0.06	**	1.02 ± 0.06	0.75 ± 0.03	**
*Bdnf* exon I	1.01 ± 0.05	0.89 ± 0.09	n.s.	1.01 ± 0.04	0.96 ± 0.09	n.s.
*tPA*	1.00 ± 0.04	0.87 ± 0.04	*	1.01 ± 0.05	1.06 ± 0.04	n.s.
**A Amygdala**						
BDNF pathway						
Total *Bdnf*	1.00 ± 0.03	1.14 ± 0.04	*	1.01 ± 0.05	1.05 ± 0.04	n.s.
*Bdnf* exon IV	1.04 ± 0.12	0.90 ± 0.08	n.s.	1.08 ± 0.14	0.70 ± 0.02	*
*Bdnf* exon I	1.05 ± 0.12	2.36 ± 0.33	**	1.10 ± 0.16	1.00 ± 0.12	n.s.
*tPA*	1.00 ± 0.03	1.46 ± 0.07	***	1.02 ± 0.08	1.33 ± 0.07	*
**B Hippocampus**						
Inflammation						
*IL-6*	1.04 ± 0.11	1.24 ± 0.15	n.s.	1.05 ± 0.14	1.13 ± 0.19	n.s.
*IL-1β*	1.04 ± 0.11	1.79 ± 0.18	**	1.02 ± 0.08	1.87 ± 0.34	*
*Calcineurin*	1.01 ± 0.05	1.03 ± 0.07	n.s.	1.02 ± 0.07	1.00 ± 0.05	n.s.
**B Amygdala**						
Inflammation						
*IL-6*	1.01 ± 0.06	2.47 ± 0.50	*	1.03 ± 0.09	2.30 ± 0.49	*
*IL-1β*	1.03 ± 0.09	3.38 ± 0.83	*	1.01 ± 0.07	2.90 ± 0.29	***
*Calcineurin*	1.00 ± 0.01	1.12 ± 0.04	*	1.00 ± 0.03	1.04 ± 0.01	n.s.

A: Effects of paroxetine on *Bdnf* and tPA mRNA levels. After chronic paroxetine treatment (~5.5 mg/kg/day for 28 days p.o.), in the hippocampus and the amygdala, no more differences were detected in total *Bdnf* mRNA level between both genotypes. *Bdnf* exon IV mRNA was less expressed in the hippocampus of both vehicle-treated (*t*_15_ = 3.94, *p* = 0.001) and paroxetine-treated (*t*_15_ = 3.44, *p* = 0.004) VGV mice. Paroxetine-treated VGV mice also presented reduced *Bdnf* exon IV mRNA expression in the amygdala (*t*_8.39_ = 2.65, *p* = 0.03). *Bdnf* exon I was not modified in the hippocampus of VGV mice. *Bdnf* exon I mRNA was highly expressed in the amygdala of vehicle-treated (*t*_12.55_ = 3.67, *p* = 0.003) VGV mice and chronic paroxetine suppressed the difference. *tPA* mRNA was less expressed in the hippocampus (*t*_15_ = 2.24, *p* = 0.0403) but significantly higher in the amygdala (*t*_11_ = 5.90, *p* = 0.0001) of VGV mice. After chronic paroxetine treatment, *tPA* mRNA level was still higher in the amygdala of VGV mice (*t*_13_ = 2.64, *p* = 0.0204) while, in the hippocampus, no more difference was detected between both genotypes. Student's *t*-test, with Welch’s correction applied if needed

B: Effects of paroxetine on inflammatory molecules mRNA levels. In the hippocampus, the level of *IL-6* mRNA expression was not different between VGV and WT mice while *IL-1β* mRNA expression was significantly higher in both vehicle-treated and paroxetine-treated VGV mice (*t*_14_ = 3.23, *p* = 0.006 and *t*_6.62_ = 2.44, *p* = 0.04, respectively). In the amygdala, *IL-6* and *IL-1β* mRNA levels were significantly higher in both vehicle-treated and paroxetine-treated VGV mice, compared to corresponding WT group (IL-6: *t*_14_ = 2.90, *p* = 0.0116 and *t*_5.34_ = 2.57, *p* = 0.04, respectively; IL-1β: *t*_5.12_ = 2.80, *p* = 0.0369 and *t*_5.55_ = 6.31, *p* = 0.001, respectively). Calcineurin mRNA level was significantly higher (*t*_9_ = 2.61, *p* = 0.0281) only in the amygdala of vehicle-treated VGV mice. No difference was detected in the hippocampus, regardless of the treatment. Student's *t*-test, with Welch’s correction applied when needed

*p < 0.05, **p < 0.01, ***p < 0.001, ****p < 0.0001 vs. the corresponding control group

Chronic paroxetine had distinct effects on *Bdnf* exon IV depending on areas: in water-treated VGV mice, exon IV mRNA was found again decreased in the hippocampus, not in the amygdala (Table 1A). However, in the amygdala, paroxetine-treated VGV mice displayed lower *Bdnf* exon IV mRNA levels compared to paroxetine-treated WT mice (Table 1A). The treatment did not change *Bdnf* exons I or IV in the hippocampus (Table 1A). The higher amygdalar *Bdnf* exon I mRNA expression observed in water-treated VGV mice was not observed after chronic paroxetine (Table 1A).

The conversion of proBDNF into the mature BDNF form is mediated by tPA. In water-treated animals, tPA mRNA expression was significantly reduced in the hippocampus of VGV mice compared to WT mice, but significantly increased in the amygdala (Table 1A). After chronic paroxetine, no difference was detected between WT and VGV mice in the hippocampus, but tPA mRNA level remained significantly higher in the amygdala (Table 1A). The alteration of tPA in the hippocampus was linked to the effect of treatment (paroxetine-treated VGV mice vs. vehicle-treated mice; *t*_15_ = 2.31, *p* = 0.0358; data not shown).

Because 5-HT2C receptor editing appears to alter neuro-inflammation^39^, classical cytokines (*IL-6*, *IL-1β*) mRNA expression levels were determined in the same brain areas of VGV mice. We also examined calcineurin, a cytokine regulated by fear extinction^51^. Amygdalar *IL-1β*, *IL-6* mRNA levels and hippocampal *IL-1β* mRNA level were increased in VGV mice, but these differences persisted after paroxetine treatment (Table 1B). Calcineurin mRNA levels was increased in the amygdala of vehicle-treated VGV mice. In contrast, this difference did not exist in paroxetine-treated VGV mice (Table 1B).

## Discussion

Increased 5-HT2CR transmission has long been involved in anxiety, and ADAR1, a 5-HT2CR editing enzyme, is increased by stress in animals and associated with suicide in patients^17,52,53^. We had previously studied VGV mice expressing only the fully edited 5-HT2CR VGV isoform, which, as a result of altered splicing event, massively express 5-HT2CR in limbic areas^24^. Because these mice display anxiety, aggressive behaviors, and strong freezing to an innately aversive stimulus^24^, we determined if they had additional features relevant to PTSD. We demonstrated that VGV mice exhibit faster fear acquisition during conditioning, extensive fear extinction deficits and fear generalization, together with alterations in brain BDNF and neuroinflammation. Our data thus suggest that VGV mice could be used as a genetic model of PTSD vulnerability as, when exposed to an important stress stimulus, they display PTSD-like behavioral and neurobiological features, some of which could be prevented by chronic paroxetine, a first line treatment of PTSD. The high freezing profile of VGV mice to an innate fear stimulus is characteristic of a state of stress sensitization^54^, similar to stress-induced 5-HT2CR activation in the amygdala triggered by behavioral stress procedures^17,55^. It was hypothesized that 5-HT2CR hyperactivity in the amygdala is central to anxiety symptoms in PTSD. Consequently, VGV mice, with their high expression of 5-HT2CR, may constitutively mimic a history of stress sensitization.

Under stress conditions, the 5-HT2CR was shown to exert a control over 5-HT neurotransmission^45^. In parallel, stress disturbs BDNF gene expression and this effect seems to be mediated at least in part, through perturbations in the serotonin signaling^32^. Inducing a reduction in BDNF also appears to intensify the anxiety-like behaviors and the stress signaling responses in mice deficient for the serotonin transporter^56^, suggesting that, in addition to their reciprocal regulatory feedback mechanism, serotonin and BDNF interact in the modulation of anxiety and stress. The BDNF Val66Met polymorphism, impacting activity-dependent secretion of BDNF, has been repeatedly described as a predisposition factor, associated in both human and animals with anxiety^57^, impaired fear extinction^58,59^, and fear generalization^37^, suggesting its involvement in PTSD^60^. VGV mice had a marked fear extinction deficit, which is consistent with the results observed after hippocampus-specific BDNF modulations^35,36^. The fear generalization observed in VGV mice is also likely an inability to properly use contextual cues to modulate fear responses via the hippocampus^37^. The increase in *Bdnf* mRNA expression in the amygdala of VGV mice is also interesting as amygdalar BDNF plays a central role for acquisition and consolidation of conditioned fear^34,49^ correlating with cue conditioned responses^61^. As already mentioned, we studied exons I and IV because they are the mains exons affected in response to stress^47^, fear^48,49^, and neuronal activity^50^. It would also be interesting to study the expression of other *Bdnf* exons. It has been shown that proBDNF, on the other hand, could disturb learning and memory^62^ and that the proteolysis of proBDNF by tPA is involved in memory formation^63^. Low levels of tPA create a deficit in long term potentiation (LTP)^64^ that has been linked to impairment in contextual and fear memory^65,66^. Moreover, stress upregulates tPA in the amygdala and this increase is linked to higher anxiety-like behaviors^67^, which is consistent with the stress sensitization-like profile of VGV mice.

Inflammation, which has important crosstalks with BDNF and serotonin^38^, has been involved in PTSD, as increased pro-inflammatory cytokines levels were detected in PTSD patients^68,69^. Extensive data indicate that inflammation affects the activity of the serotonergic system, notably the activity of the serotonin transporter (for review, see ref. ^38^). More interestingly, inflammatory cytokines were shown to induce an increase in 5-HT2CR editing levels^39^. Aside from the perturbations that an inflammatory state could induce in the mutually regulating brain balance between serotonergic and BDNF signaling, direct links between inflammation, memory, or neuroplasticity involving BDNF were also found^70–72^ as well as between inflammation and memory relevant to fear processing^73,74^. Calcineurin was also involved in fear memory^75,76^ and an enhanced calcineurin activity in the amygdala was linked to fear extinction deficits^51^. Finally, neuro-inflammation also impairs contextual discrimination, without impacting other hippocampal-dependent tasks such as spatial memory^77,78^. VGV mice seem to constitutively present a pro-inflammatory status, indicated by the overexpression of *IL-1β, IL-6*, and calcineurin mRNA. We could hypothesize that the augmented expression of 5-HT2CR in VGV mice may trigger, through Gq-protein-mediated PLC activity, an increased Ca^2+^ mobilization in affected neurons and a high intracellular Ca^2+^ level is known to induce neuroinflammation by activating caspase-1, an enzyme responsible for the maturation of IL-1β^79^. Additionally, the 5-HT2CRs via Gq-protein induce PLA2 activity is also well-known to mediate inflammatory responses^80^. The neuroinflammation observed here in VGV mice is therefore consistent with the literature.

**Fig. 5:**
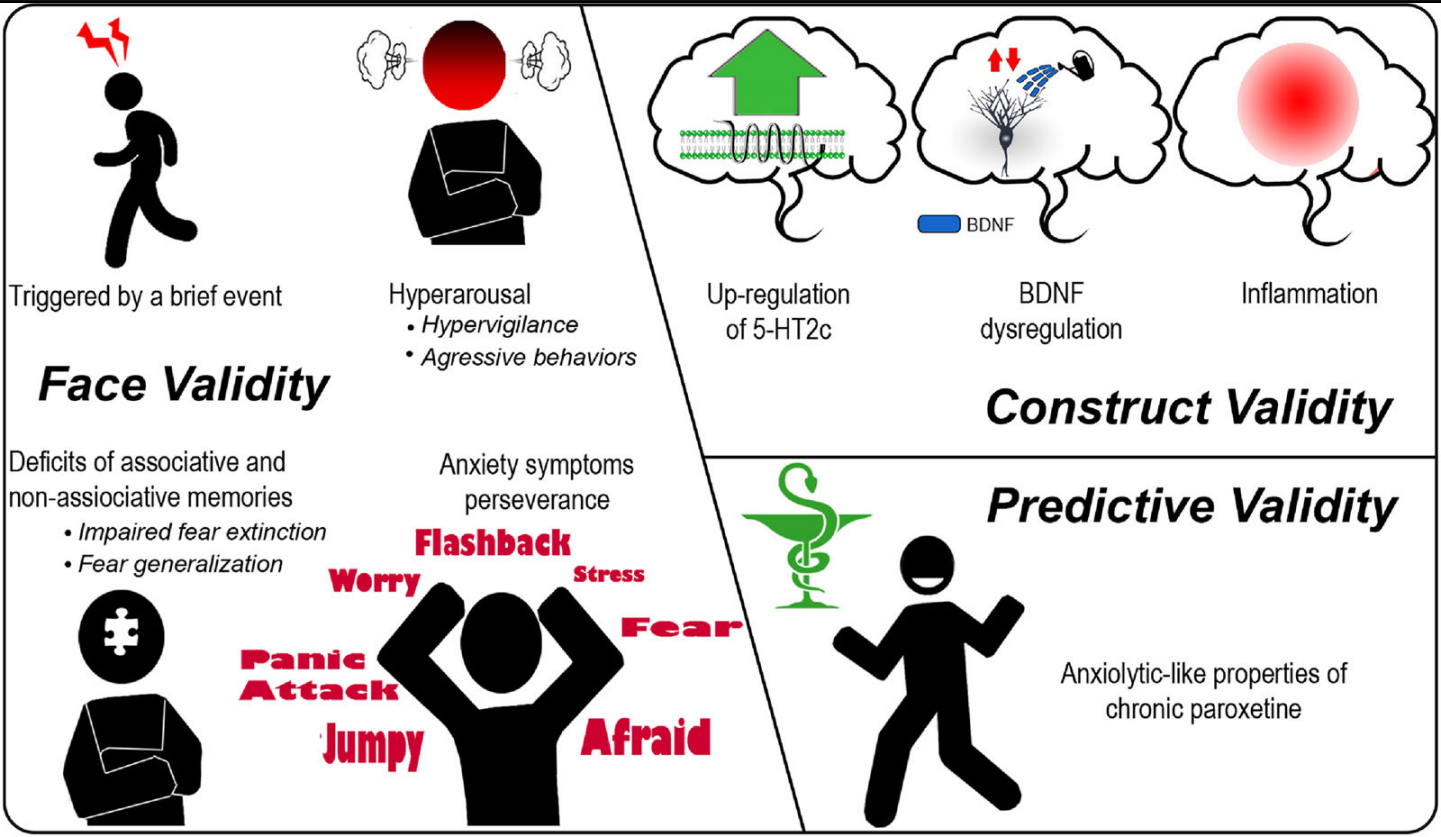
Pros and cons about the validity of VGV mice as a model of vulnerability to PTSD. Assessment of current data in view of a PTSD validity criteria checklist^93^. **a** Face validity: both associative and non-associative memories are part of the traumatic fear state, and these two components need to be inhibited to treat PTSD. VGV mice displayed both abnormal associative memories (impaired fear extinction) (Fig. 1d, e) and non-associative memories (fear sensitization) (Fig. 1c). Human PTSD is most often triggered by a brief event and the PTSD-like behavioral state of VGV mice can be induced by a relatively brief aversive stressor. Another criterion is symptom perseverance: VGV mice’s fear-related dysregulations persisted weeks after conditioning and did not spontaneously go into extinction, even after numerous context or cue presentations (Fig. S2A). VGV mice also presented exaggerated fear responses, that is hypervigilance, to trauma-related cues (CS) (Fig. 1b) as well as to innately aversive stimuli (Fig. S5)^24,27^. **b** Construct validity: *Bdnf* mRNA exons and *tPA* mRNA dysregulations were found in the hippocampus, the amygdala, and the frontal cortex of VGV mice (Fig. 4 and Table 1A). Such dysregulations had previously been suggested to account for memory processes, synaptic plasticity, and neuronal activity impairments in patients. Deficit in fear extinction correlates with lower hippocampal activation^97^ and hyperactivation of the amygdala^98^. An increase in pro-inflammatory cytokines IL-1β and IL-6 mRNA levels was also found in the hippocampus and the amygdala of VGV mice (Table 1B). Inflammation appears to be involved in both the pathophysiology of PTSD^95,99^ and in PTSD vulnerability^96^. 5-HT2CR expression is upregulated in the VGV mice and these receptors are known to be associated with vulnerability to mood disorders in human^100–102^. One limitation of the present genetic model is the extent of 5-HT2CR upregulation in these mice. **c** Predictive validity: paroxetine is the first-line pharmacological treatment of PTSD. Chronic paroxetine produces anxiolytic effects in human PTSD as well as in VGV mice. Chronic paroxetine is given here either before the fear conditioning or as a post-traumatic treatment, with similar results (Fig. 2). The putative improved therapeutic effect of a paroxetine treatment starting prior to exposure therapy still needs to be assessed in human

Since chronic paroxetine is known to desensitize 5-HT2CRs^40^, we first used this treatment to reduce the mRNA editing-mediated 5-HT2CRs overexpression phenotype of VGV mice and examine its effects on the behavioral and neurobiological changes found in VGV mice. Chronic paroxetine reduced generalization, maybe by normalizing hippocampal BDNF and neuronal excitability^81^. The increased *Bdnf* in the amygdala of VGV mice was successfully prevented by chronic paroxetine, consistently with its anxiolytic effect^82^, with data about 5-HT2CR desensitization occurring, at the behavioral, neurochemical, and cell-signaling levels, and after 5-HT reuptake carrier inactivation^40,45^. The paroxetine-induced 5-HT2CR desensitization led to reduced freezing in VGV mice during both conditioning and tone testing via an attenuation of non-associative fear sensitization. However, trauma-associated fear memory can be subdivided in two forms of learning: associative memories, directly resulting from CS–US pairing during the conditioning process, and non-associative memories, involving anxiety-like fear sensitization^83,84^. Both of these memory components (present in VGV mice; Fig. 5) should be inhibited to allow successfully overcoming a traumatic event and preventing later fear reinstatement. Additionally, except for amygdalar calcineurin mRNA levels, neuroinflammation was not prevented by chronic paroxetine. Note that calcineurin can directly interact with the serotonin transporter^85^, a mechanism which may underlie the positive effect of paroxetine on calcineurin in VGV mice. In a previous study, a tricyclic antidepressant drug was better than paroxetine to decrease brain inflammation factors^86^. The present murine model could thus be used to decipher how to best treat anxiety-associated inflammation. It has been argued that SSRI’s anti-inflammatory effect is not sufficient, justifying the necessity of investigating alternative agents with clear anti-inflammatory properties, such as glucocorticoids that additionally have a therapeutic effect on other PTSD symptoms^87^.

Here, our two different experimental timelines for the chronic paroxetine treatment produced the same behavioral outcomes in VGV mice. However, one has to keep in mind that the rationale for the first chronic treatment prior to shock exposure was to determine the effects of 5-HT2CR desensitization, not to mimic a clinical use of paroxetine as a prophylactic treatment. Indeed, as previously observed in rats^5^, we observed that chronic paroxetine tends to impair fear extinction in WT mice, which argues against using paroxetine as a prophylactic treatment.

Chronic paroxetine was ineffective in restoring the extinction process in VGV mice. We thus decided to assess whether blocking 5-HT2CR would have an effect. Agomelatine, which is a relatively weak and non-selective 5-HT2CR antagonist, did not produce a significant effect, while the hyperactivity produced by the potent and selective 5-HT2CR antagonist SB242084^46^, precluded any conclusion. Interestingly, it has been demonstrated that the binding profile of agomelatine is not modified by the level of edition of the 5-HT2CR isoforms^88^, which is a factor to consider when targeting directly the 5-HT2CR. In turn, acute paroxetine did trigger within session fear extinction in VGV mice. Since the half-life of paroxetine in the mice brain was estimated at 2 h^89^ and its metabolites are inactive^90^, this strongly suggests that the effects observed at Day 3 are not the effect of some residual paroxetine molecules administered on the previous day, but rather are long-term consequences of some processes initiated on Day 2. The injection at Day 2 might have produced therapeutic-like effects by producing a surge in extracellular 5-HT, triggering somatodendritic 5-HT_1A_ and terminal 5-HT_1B_ autoreceptors activation, thereby decreasing neuronal firing and intrasynaptic 5-HT availability. Alternatively, acute paroxetine-induced surge in extracellular 5-HT might have restored fear extinction by activating post-synaptic 5-HT receptors, such as the 5-HT2AR subtype^91^.

Currently, neither pharmacological nor behavioral approaches are completely effective as there are non-responders to either approach. Data reporting the effects of combining one of the approved SSRI with an exposure therapy are rare and rather controversial. In a clinically relevant perspective (Fig. 5), the latter data suggest that combining these two types of therapies could present beneficial outcomes, but with a precise timeline of SSRI administration. More precisely, it suggests that the best period to initiate a SSRI treatment in PTSD patients is at the very beginning of an exposure therapy, as it could initially facilitate fear extinction memory acquisition, while prolongation of treatment up to chronicity can provide additional anxiolytic effects. However, caution has to be taken as it has been extensively described that acute SSRI treatments can trigger anxiogenic effects. In any case, chronic treatment has advantages compared to acute treatment, as acute SSRI treatment presents several well-described adverse effects (sexual and gastro-intestinal dysregulations, perturbations of appetite and weight, increased anxiety, among others) that nevertheless tend to disappear with chronic treatment. The idea that a specific timeline of treatment administration could provide beneficial effects was also recently studied with agomelatine. A single dose of this drug administered rapidly in the aftermath of a traumatic event seems to reduce the development of PTSD-like behavioral responses and the hippocampal stress-induced damages^92^.

The putative “face validity” of the present model should regroup pre-trauma cognitive vulnerability factors and PTSD-like symptoms in line with the “dual-branch hypothesis of PTSD”^93^. Accordingly, a PTSD model needs to combine both the memory- and stress-related processes. The characteristics of the PTSD-like symptoms of VGV model are in line with this criterion, as detailed in the Fig. 5. We hypothesized earlier that VGV mice could constitutively model a state of stress sensitization. This characteristic could mimic a reported vulnerability factor, the looming cognitive style^94^. The increase responses of VGV mice’s response to innate fear stimuli (initial reactions to foot-shocks and ultrasonic stimulus) could thus represent a behavioral manifestation of a fear sensitization in these animals. Regarding the “construct validity” criterion (Fig. 5), while it is not yet known why certain individuals develop PTSD after a traumatic event while others do not, it remains worth assessing this validity criterion around some of the most accepted hypotheses concerning PTSD pathophysiology, and numerous authors suggest relations between BDNF, inflammation, and PTSD predisposition^95,96^.

Overall, this study shows that VGV mice may constitute an interesting model of PTSD predisposition. These mice present important enhancements of both innate and conditioned fear. The present model has, nevertheless, limitations common to most genetic models, since the major alterations in serotonergic transmission, predisposing to PTSD-like behaviors, are triggered during development. Further studies should also investigate whether the PTSD-like profile of adult VGV mice can be reversed by vector-induced reduction of 5-HT2CR in the amygdala or by modulating amygdalar and hippocampal BDNF–TrkB pathway. Moreover, the genetic VGV model provides opportunities to further understand the role of the serotonin signaling on both the psychophysiological and biological correlates of PTSD. Finally, because it readily mimics an intense state of stress-sensitization and neuroinflammation, it offers new perspectives to quickly and effectively screen innovative drugs for PTSD.

## Supplementary information

Supplementary Methods & Material and Results.
