# Supplementary material for: Increased 5-HT2C receptor editing predisposes to PTSD-like behaviors and alters BDNF and cytokines signaling
Source: Transl Psychiatry. 2019 Feb 21;9:100. doi: 10.1038/s41398-019-0431-8 (PMC6384909; doi:10.1038/s41398-019-0431-8)
Supplement: Supplementary file 1 — Supplementary Methods & Material and Results. [file 41398_2019_431_MOESM1_ESM.docx]

**Supplementary Methods & Material**

*Animals*

Mice were kept under standard laboratory conditions (12h light/dark cycle, room temperature 21±1°C) with free access to food and water. They were housed 2-6 per cage (29x18 cm) or during chronic treatments, only 1 per cage in order to assess liquid consumption. Mice expressing VGV 5-HTR2C were generated and backcrossed for over 10 generations into the C57BL/6J genetic background. All animals, regardless of the sex, were 10-week-old at the beginning of each experiment. Each experiment was performed with independent groups of mice. Basal fear conditioning required n=8-10 animals per groups, while for acute paroxetine treatment results were assessed with n=10-14 in each group of treatment and for chronic paroxetine procedure followed by fear conditioning, results were solidified with n=10-12 VGV mice per group and n=16-17 WT mice per group of treatment. Innate fear analyses required n=10-14 animals per group. Molecular mRNA quantification was performed on independent groups of animals that never underwent behavioral training, to exclude environmental effects, with n=6-10 animals per group. Supplemental behavioral procedures were assessed with n=5-6 animals per groups, except for the Barnes maze experiment that was performed with n=7-9 animals per group.

*Conditioned fear procedure:*

Apparatus: A computer-controlled fear conditioning system (MedAssociates, St-Albans, VT, USA) was used. Conditioning took place in a lit transparent conditioning chamber (25x20 cm) with metal grid floor surrounded by a sound-attenuating cubicle. Extinction was conducted in another room, with lights off, using housing cages mounted with lid and speakers and in front of an infrared light panel.

For the fear conditioning procedure, on Day 1, mice were placed in the conditioning chamber and after a 3 min baseline period, they received 6 times an auditory conditional stimulus (CS; 30s, 2.5 kHz, 85dB) immediately followed by the unconditioned stimulus (US; 2s, 0.5mA foot-shock, inter-trial intervals 2 min). Then, cued extinction was performed. On Day 2, for experimental designs 1 & 3, or Day 30, for experimental design 2 (Fig.2; Fig.3), mice received 20 exposures to the same tone (30s, 2.5 kHz, 85dB; inter-trial intervals 5s) in a new context to assess CS-induced fear and its extinction. On Fig.1, and Fig.3, the same procedure was repeated on Day 3, to determine consolidation of the fear extinction memory. Both intra-session and inter-session extinction indexes were defined, respectively, as (Freezing at CS1) – (Freezing at CS6) and (Day 2 freezing at CS1) – (Day 3 freezing at CS1), to compare between WT and VGV mice the amount of extinction acquired on the same day or consolidated on the second extinction day.

Behaviors were monitored by a video camera. Freezing, defined as total lack of movement, except respiration, was scored every 2s by an observer blind to genotype and treatment during the acquisition phase. Freezing during extinction was recorded by an infrared filtered camera associated with a movement detection software (Viewpoint, Lyon, France). Preliminary experiments indicated that manual and automatic scorings were consistent.

*Innate fear procedure*

Mice were tested for their innate fear reactions to a train of ultrasonic stimuli in their home cage, using 100-ms frequency sweeps of 17–20 kHz, 85 dB, alternately 2 s ON and then 2 s OFF for 1 min, after a 3-min baseline period. Ultrasounds trigger two behavioral reactions: flight reactions triggered during the ON periods and freezing reactions, which was quantified by sampling events of complete immobility (except breathing) every 2 s during a 1-min post-stimulus period. These reactions are innate because they appear immediately after the first 2 s ultrasound stimulus. Ultrasounds were provided using a speaker attached to a lid placed on the home cage and data were gathered by a video camera.

*Barnes maze*

The apparatus is a white circular platform, 80 cm in diameter, elevated 50 cm above the floor. Eighteen 5 cm-diameter holes are evenly spaced around the platform edge. A removable escape box (8 × 5 × 5 cm) can be placed under any of these holes. Constant visual cues of distinct colors and shapes are placed on the walls around the maze. All sessions were performed under bright light on a wet platform to increase the mouse aversion for the platform. Sessions were recorded using a video-tracking system (Viewpoint, Lyon, France). The day before the learning sessions, mice were habituated to the apparatus. The mice were trained for 4 days with 3 sessions per day. The reversal learning was performed during 4 days with 3 sessions per day with the escape box moved 120° from its original position. The number of errors and the latency before finding the escape box were measured for both training sessions and reversal learning sessions. The reversal probe test was conducted immediately after the last reversal learning session. The escape box was removed and the mouse was allowed to explore the maze for 1 min and the time spent in each sextant was quantified.

*Quantification of RNA levels by RT–qPCR*

Tissue samples were quickly removed and frozen in liquid nitrogen. Each analyzed structure (prefrontal cortex, amygdala and hippocampus) was taken as a whole, without considering sub-areas. Total mRNA was extracted using TRI Reagent (Ambion, Applied Biosystems, Courtaboeuf, France), following manufacturer's instructions. Reverse transcription was performed using the following protocol: 25°C, 10 min; 37°C, 2 h; 85°C, 5 s. PCR amplifications were performed with the following cycling protocol: 95°C for 3 min followed by 40 cycles of 95°C for 15 s, 60°C for 30 s and 72°C for 30 s. Analysis of Bdnf expression was performed according to the nomenclature of Aid et al. Primers: ACTB, forward: 5’-CCACCATGTACCCAGGCATT-3’, reverse: 5’- CGGACTCATCGTACTCCTGC-3’; Total BDNF, 5’-TGCAGGGGCATAGACAAAAG-3’, reverse: 5’-TGAATCGCCAGCCAATTCTC-3’; BDNF I, forward: 5’-GTGTGACCTGAGCAGTGGGCAAAGGA-3’, reverse: 5’-GAAGTGTACAAGTCCGCGTCCTTA-3’; BDNF IV, forward: 5’-CTCTGCCTAGATCAAATGGAGCTTC-3’, reverse: 5’-GAAGTGTACAAGTCCGCGTCCTTA; tPA, forward: 5’-AGGCAACCAAGACCTCCAC-3’, reverse: 5’-TGTAGACCCCAGGCACATC-3’; CRH, forward: 5’-CCTTGAATTTCTTGCAGCCG-3’, reverse: 5’-GGACTTCTGTTGAGATTCCCC-3’; IL-6, forward: 5’-AGTTGCCTTCTTGGGACTGA-3’, reverse: 5’-TCCACGATTTCCCAGAGAAC-3’; IL-1β, forward: 5’-GCCCATCCTCTGTGACTCAT-3’, reverse: 5’-AGGCCACAGGTATTTTGTCG-3’; Calcineurin, forward: 5’-CCACAGGGATGTTGCCTAGTG-3’, reverse: 5’-GTCCCGTGGTTCTCAGTGGTA-3’. Gene expression was normalized by reference to the housekeeping gene β-actin and analyzed using the 2ΔΔCT (Delta-Delta Comparative Threshold) method, described by Livak and Schmittigen (2001).

*Statistical analysis of qRT-PCR results*

qRT-PCR results are analysed and presented using the 2ΔΔCt described by Livak and Schmittigen (2001). This method generates groups that are not independent, therefore we do not meet the criteria for an ANOVA. Consequently, unpaired two-tailed Student’s t-tests were used for the two-groups comparisons, with Welch’s correction if needed.

Each result presented in this article were replicated independently at least two times in the laboratory, with consistent outcomes, except for the Barnes Maze which was performed only once.

**Supplementary results**


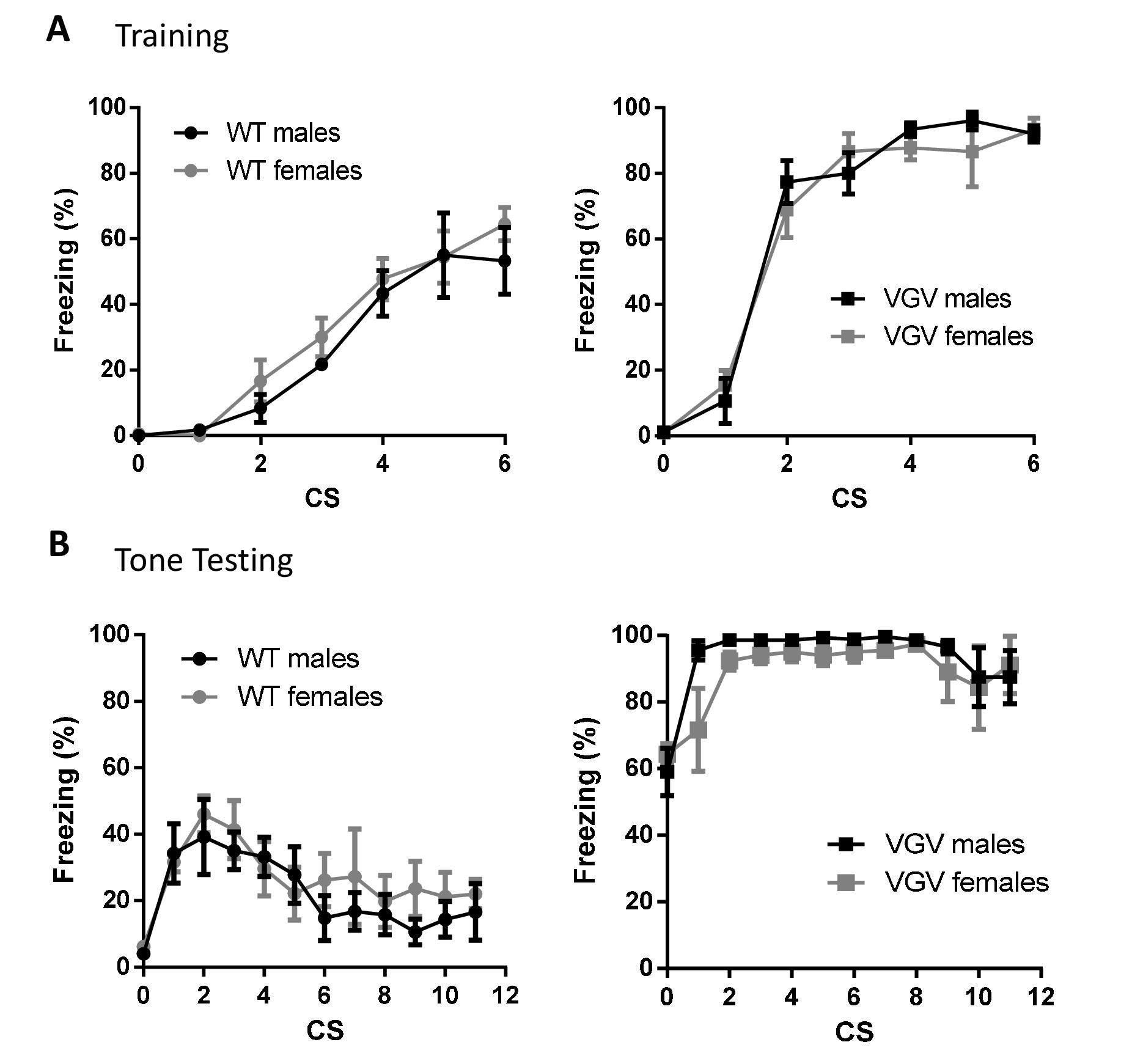


**Figure S1: No gender difference in the conditioned fear behavioral profile of VGV mice.**

(**A)** Female mice presented the same fear acquisition profile compared to male mice, in accordance with their genotype (WT: no effect of Gender [F (1, 8) = 1.64, p=0.2], a significant effect of Time [F (6, 48) = 33.84, p < 0.0001]; VGV: no effect of Gender [F (1, 9) = 0.0864, p=0.8], a significant effect of Time [F (6, 54) = 144.7, p < 0.0001]).

(**B**) Female VGV mice displayed similar fear extinction and fear generalization deficits compared to male VGV mice (WT: no effect of Gender [F (1, 8) = 0.6643, p=0.4386], a significant effect of Time [F (12, 96) = 4.512, p < 0.0001]; VGV: no effect of Gender [F (1, 10) = 1.169, p=0.3049], a significant effect of Time [F (11, 110) = 9.14, p < 0.0001]).


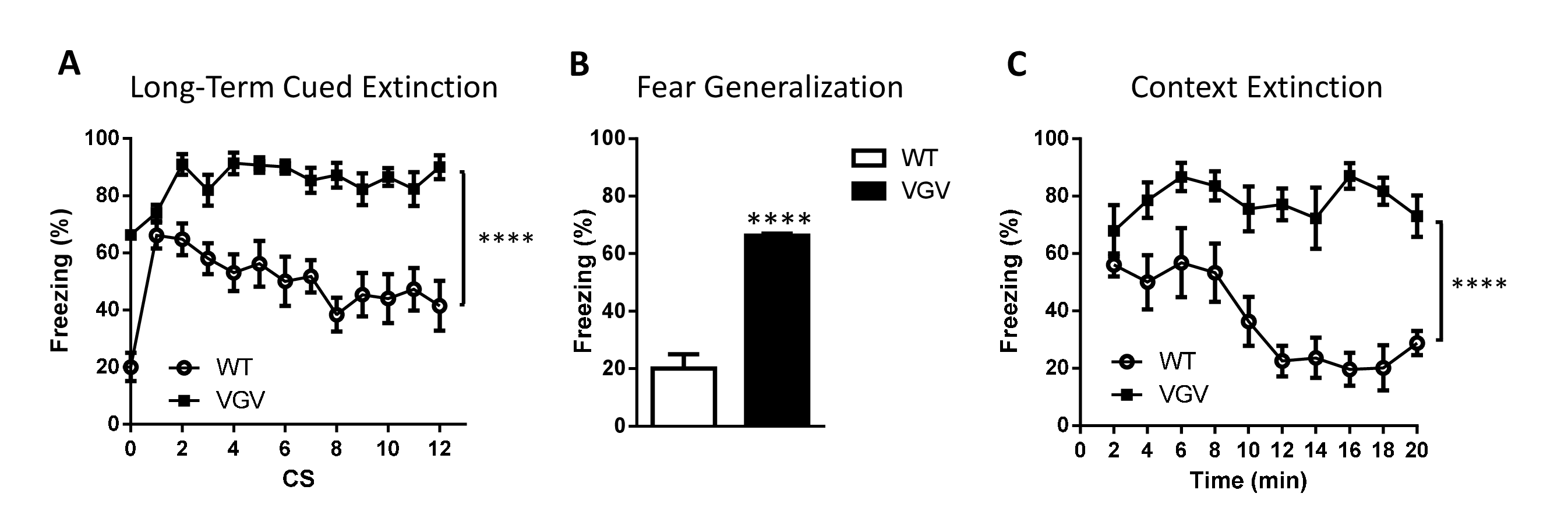


**Figure S2: Long lasting deficits in cue fear extinction, fear generalization and contextual fear extinction in VGV mice.** After a period of 4 weeks following the conditioning session, WT and VGV mice went through the extinction session. VGV mice still presented a very high freezing response and a fear extinction deficit (**A**) and generalization of contextual fear (**B**).

For the extinction session, two-way ANOVA with repeated measures indicated a significant effect of Genotype [F (1, 16) = 36.14, p < 0.0001], Time [F (12, 192) = 8.92, p < 0.0001] and an interaction between those two factors [F (12, 192) = 4.16, p < 0.0001]. For the fear generalization, Student’s t-test between WT and VGV indicated a highly significant difference (t_8.227_=9.21, p < 0.0001).

(**C**) VGV mice showed a deficit of contextual fear extinction when re-exposed to the conditioning context. Two-way ANOVA with repeated measures indicated a significant effect of Genotype [F (1, 11) = 38.5, p < 0.0001], Time [F (9, 99) = 3.85, p= 0.0003] and an interaction between those two factors [F (9, 99) = 4.05, p=0.0002].


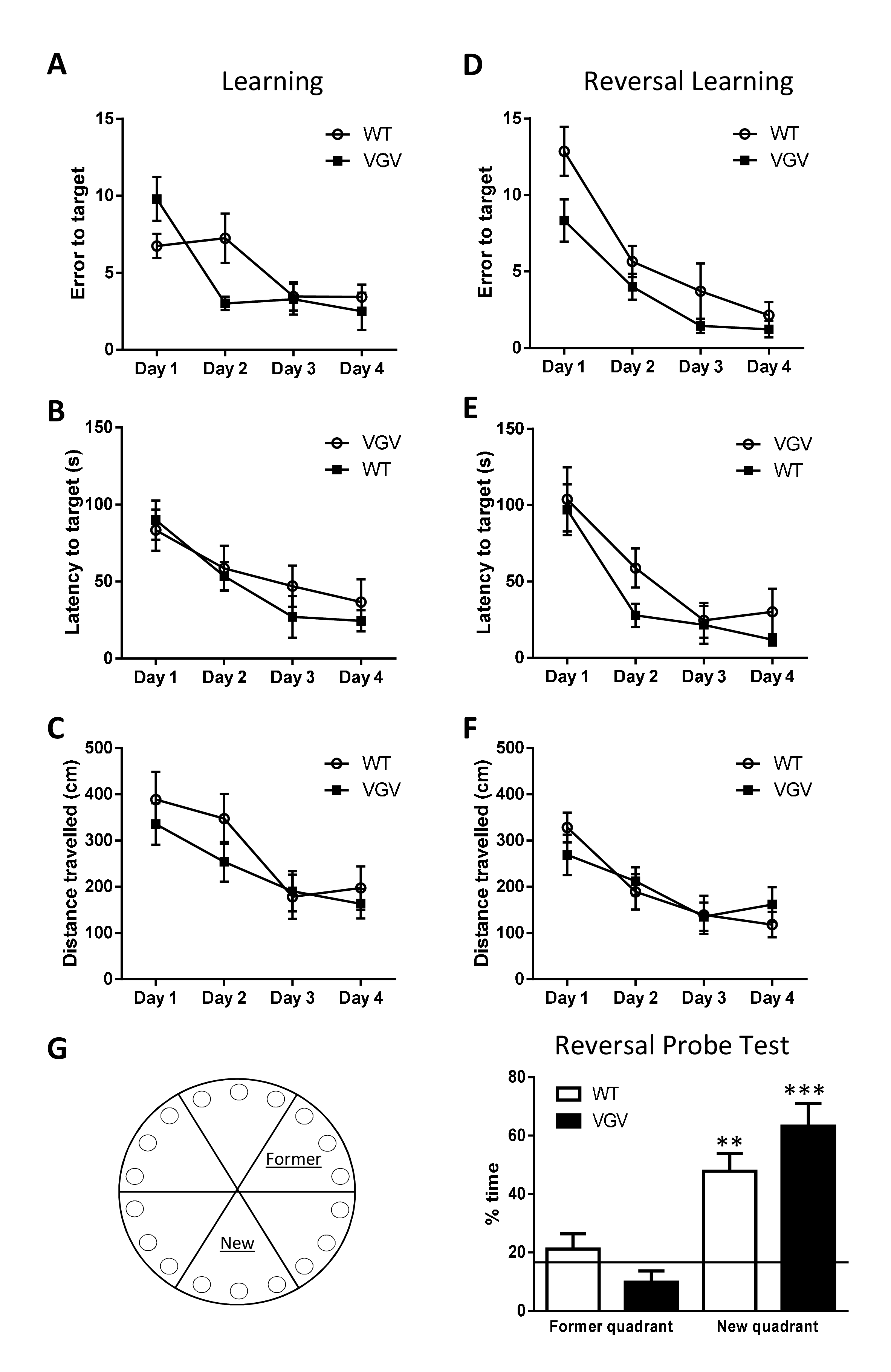


**Figure S3:** **Spatial memory and cognitive flexibility in VGV mice.** To address spatial memory and cognitive flexibility in VGV mice, animals were trained to find the location of an escape box on a circular platform and the number of errors was assessed as a measure of spatial memory acquisition. Data are the mean of the 3 daily sessions.

(**A**) Both WT and VGV mice optimally learned the location of the escape box in terms of the number of errors. The graph displays the average number of error per day of training. Two-way ANOVA indicated no effect of Genotype but a significant effect of Time [F (3, 42) = 16.67, P < 0.0001]. There were no changes either in terms of latency to reach the target (**B**) [a significant effect of time (F (3, 42) = 9.54, p< 0.0001) and no effect of Genotype], and (**C**) in terms of distance travelled [significant effect of Time (F (3, 42) = 12.20, p< 0.0001) but no effect of Genotype].

(**D**) To assess cognitive flexibility, the escape box was moved 120°. Both genotypes performed equally during the reversal learning session [significant effect of Time, F (3, 42) = 32.53, P < 0.0001] (**E**). There were no changes either in latency to reach the target [a significant effect of Time (F (3, 42) = 24.14, p< 0.0001) and no effect of Genotype] (**F**) and distance travelled [significant effect of Time (F (3, 42) = 11.82, p< 0.0001), but no effect of Genotype].

(**G**) For the reversal probe test, the escape box was removed and mice were allowed to explore the platform. The percentages of time spent in the newly learned quadrant and in the former quadrant, corresponding to the original escape box location, were analyzed. VGV mice did not display extinction deficits toward the former target location. A two-way ANOVA indicated a significant effect for the quadrant location (F (1, 24) = 45.83, p< 0.0001) but no effect of Genotype. The animals spent more time in the newly learn sextant location than random (vs. 16.67 % = the theoretical percentage of time spent randomly exploring each quadrant). Data were analyzed using one sample t-test. Data shown are mean ± s.e.m. **p<0.01, ***p<0.001.


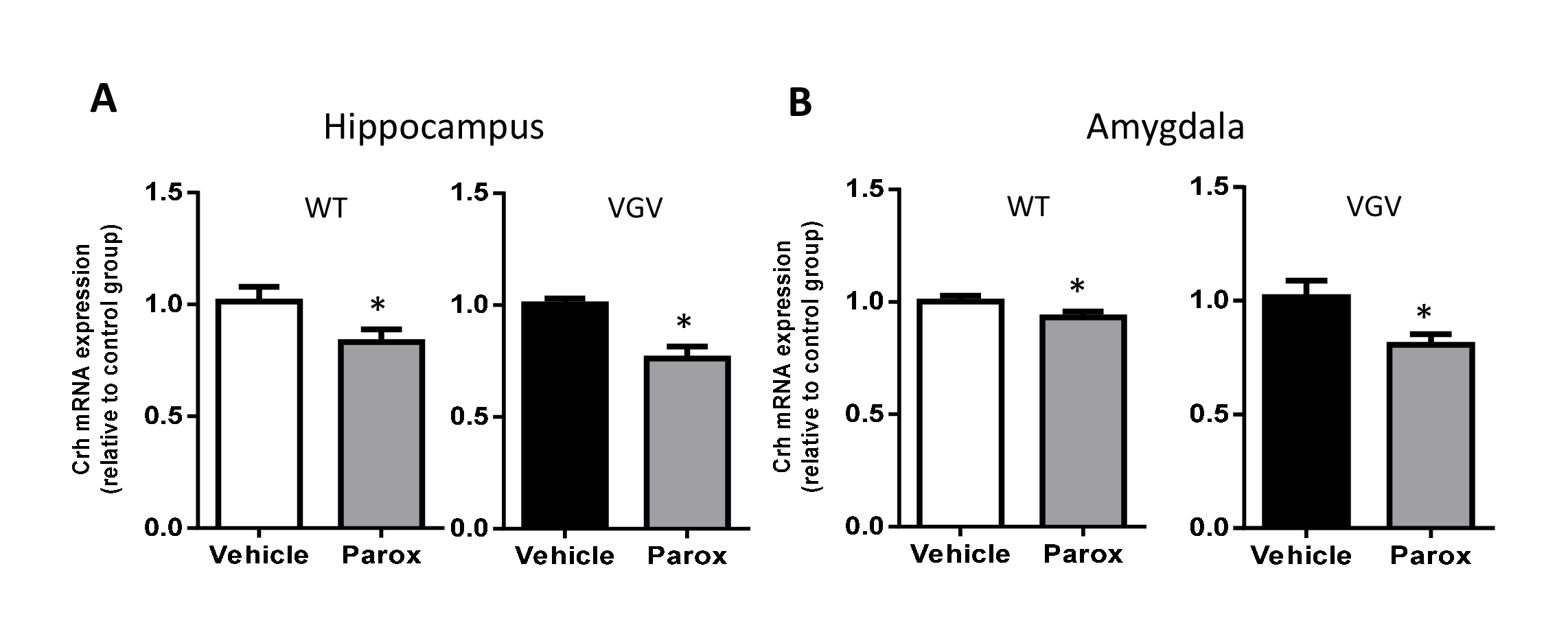


**Figure S4: Assessment of drug delivery in the brain using the antidepressant-induced decrease of CRH mRNA assay.** Chronic paroxetine treatment administered to the animals via the drinking water (~ 5.5 mg/kg/day for 28 days) significantly decreased the Crh mRNA expression in (**A**) the hippocampus (WT: t_13_= 2.061, p= 0.03; VGV: t_14_= 4.308, p= 0.0004) and (**B**) the amygdala (WT: t_14_= 1.972, p= 0.0344; VGV: t_13_= 2.419, p= 0.0155) of both genotypes. Student t-test, *p<0.05.


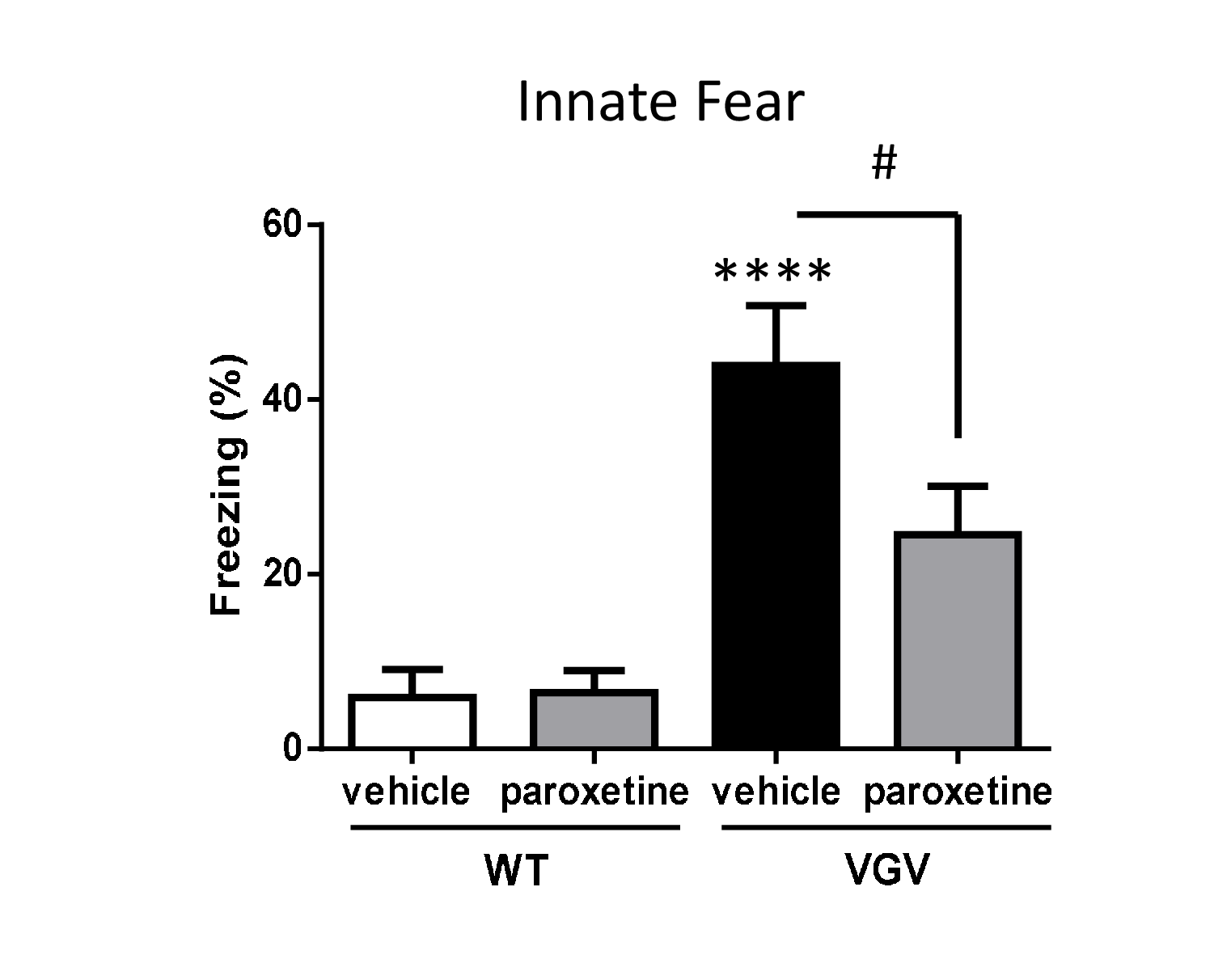


**Figure S5: Effects of chronic paroxetine on innate fear in VGV mice.** Innate fear corresponds to the immediate expression of reflex-like defensive behaviors, here freezing, generated by a stimulus not associated with a previous aversive event. VGV mice display high innate fear-induced freezing during the 1-min post-ultrasounds period and this higher freezing was reduced by chronic paroxetine. ANOVA indicated a significant effect of Genotype [F (1, 44) = 38.99, p < 0.0001], of Treatment [F (1, 44) = 4.39, p = 0.04] and an interaction between factors [F (1, 44) = 4.91, p = 0.03].


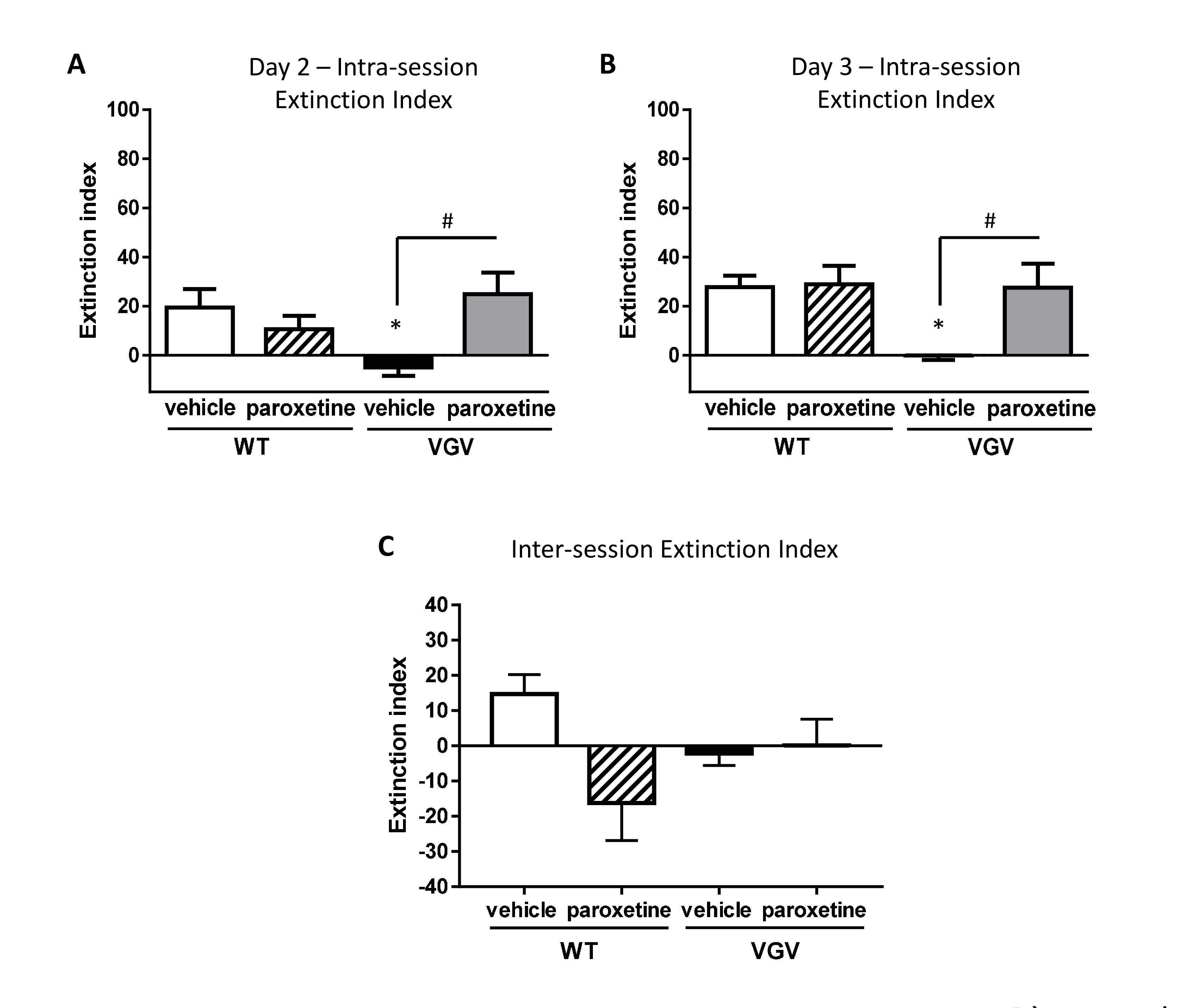


**Figure S6: Effects of acute paroxetine on the extinction index.**

(**A**) On Day 2, acute paroxetine (16 mg/kg, i.p., 30 min before the first extinction session) restored extinction in VGV mice, as observed with the increased intra-session extinction index, defined as (Freezing at CS1) – (Freezing at CS6). (Interaction between the factors genotype and treatment [F(1, 40)=8.25, p=0.0065]; Bonferroni post-hoc test indicating a significant difference between WT Vehicle and VGV Vehicle groups as well as between VGV Vehicle and VGV Paroxetine groups).

(**B**) On Day 3, this effect is conserved (effect of Treatment [F (1, 40) =4.82, p= 0.034], effect of Genotype [F (1, 40) = 4.98, p=0.031], interaction between factors [F (1, 40) = 4.05, p= 0.050]; Bonferroni post-hoc test indicating a significant difference between WT Vehicle and VGV Vehicle groups as well as between VGV Vehicle and VGV Paroxetine groups).

(**C**) However, by analyzing the inter-session extinction index defined as (Day 2 freezing at CS1) – (Day 3 freezing at CS1), acute paroxetine did not reverse the lack of extinction retention in VGV mice. *p<0.05 vs WT vehicle; #p<0.05 vs VGV vehicle.


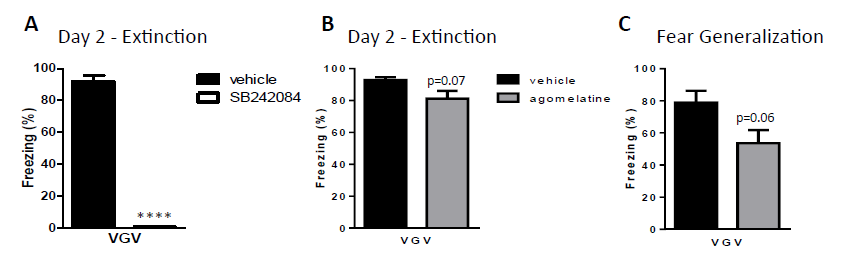


**Figure S7: Effects of 5-HT2C antagonists on conditioned fear in VGV mice.**

(**A**) Acute injection of SB242084 (1mg/kg, *i.p*, 30 min prior to the session) totally suppress freezing in VGV mice (t4.14=25.81, p<0.0001).

(**B**) After acute administration of agomelatine (50 mg/kg, *i.p*., 30 min prior to the session), there was only a trend toward decrease freezing during the extinction session (t_6_=2.249, p= 0.07).

(**C**) The expression of contextual fear generalization also tended to be reduced in VGV mice (t_6_=2.299, p= 0.06). Data are mean +/- s.e.m, analyzed with the Student t-test (with Welch’s correction when needed).
